# Effects of sarcosine and N, N-dimethylglycine on NMDA receptor-mediated excitatory field potentials

**DOI:** 10.1186/s12929-016-0314-8

**Published:** 2017-02-28

**Authors:** Mei-Yi Lee, Yi-Ruu Lin, Yi-Shu Tu, Yufeng Jane Tseng, Ming-Huan Chan, Hwei-Hsien Chen

**Affiliations:** 1 Master/PhD Program in Pharmacology and Toxicology, Tzu Chi University, 701, Section 3, Chung-Yang Road, Hualien, 97004 Taiwan; 2 Institute of Biomedical Electronics and Bioinformatics, National Taiwan University, 1, Sec. 4, Roosevelt Rd., Taipei, 10617 Taiwan; 3 Department of Computer Science and Information Engineering, National Taiwan University, 1, Sec. 4, Roosevelt Rd., Taipei, 10617 Taiwan; 4 Institute of Neuroscience, National Chengchi University, 64, Sec. 2, ZhiNan Road, Wenshan District Taipei City, 11605 Taiwan; 5 Research Center for Mind, Brain, and Learning, National Chengchi University, 64, Sec. 2, ZhiNan Road, Wenshan District Taipei City, 11605 Taiwan; 6 Center for Neuropsychiatric Research, National Health Research Institutes, 35 Keyan Road, Zhunan, Miaoli County 35053 Taiwan

**Keywords:** N-methylglycine, Glycine binding site, D-serine, D-cycloserine, 7-chlorokynurenate

## Abstract

**Background:**

Sarcosine, a glycine transporter type 1 inhibitor and an N-methyl-D-aspartate (NMDA) receptor co-agonist at the glycine binding site, potentiates NMDA receptor function. Structurally similar to sarcosine, N,N-dimethylglycine (DMG) is also N-methyl glycine-derivative amino acid and commonly used as a dietary supplement. The present study compared the effects of sarcosine and DMG on NMDA receptor-mediated excitatory field potentials (EFPs) in mouse medial prefrontal cortex brain slices using a multi-electrode array system.

**Results:**

Glycine, sarcosine and DMG alone did not alter the NMDA receptor-mediated EFPs, but in combination with glutamate, glycine and its N-methyl derivatives significantly increased the frequency and amplitude of EFPs. The enhancing effects of glycine analogs in combination with glutamate on EFPs were remarkably reduced by the glycine binding site antagonist 7-chlorokynurenate (7-CK). However, DMG, but not sarcosine, reduced the frequency and amplitude of EFPs elicited by co-application of glutamate plus glycine. D-cycloserine, a partial agonist at the glycine binding site on NMDA receptors, affected EFPs in a similar manner to DMG. Furthermore, DMG, but not sarcosine, reduced the frequencies and amplitudes of EFPs elicited by glutamate plus D-serine, another endogenous ligand for glycine binding site.

**Conclusions:**

These findings suggest that sarcosine acts as a full agonist, yet DMG is a partial agonist at glycine binding site of NMDA receptors. The molecular docking analysis indicated that the interactions of glycine, sarcosine, and DMG to NMDA receptors are highly similar, supporting that the glycine binding site of NMDA receptors is a critical target site for sarcosine and DMG.

**Electronic supplementary material:**

The online version of this article (doi:10.1186/s12929-016-0314-8) contains supplementary material, which is available to authorized users.

## Background

Derivatives of glycine, including N-methylglycine (sarcosine) and N, N-dimethylglycine (DMG) are important intermediates in the metabolism of choline to glycine [30, 40]. Sarcosine and DMG are widely distributed in food and used as dietary supplements. These two structurally similar glycine derivatives were reported to have pharmacological activities in the central nervous system.

Sarcosine is a competitive glycine transporter type-l inhibitor [16], an N-methyl-D-aspartic acid (NMDA) receptor co-agonist [45], and a glycine receptor agonist [46]. Based on its enhancing effects on NMDA receptors, sarcosine has been studied for its efficacy in ameliorating negative and cognitive symptoms in patients with schizophrenia, showing promising results [18, 21, 33, 35]. In addition, sarcosine can improve depression-like behaviors in rodent models and in human depression [18]. The toluene-induced behavioral aberrations were also attenuated by sarcosine [4].

DMG, consisting of one more methyl group than sarcosine, has been reported to decrease oxidative stress [36], to improve immune responses [15] and to be promoted as an athletic performance enhancer [34]. DMG also acts on central nervous system since it exhibits anticonvulsant activity in animal models [11]. Recently, it has been reported that DMG has antidepressant-like effect and reduces the ketamine-induced psychotomimetic behaviors in mice [24]. Furthermore, DMG has been tested as a supplement for patients with autism or pervasive developmental disorder [1, 3, 19, 43], although the results from these clinical studies are controversial.

Opening of the NMDA receptor channel complex requires occupation of its glutamate binding site by glutamate and its glycine binding site by glycine or D-serine [41]. Thus, glycine is not only a glycine receptor agonist, but also a co-agonist of NMDA receptors. In addition to inhibiting the glycine transporter 1 and increasing the ambient glycine concentration, sarcosine, simply the glycine molecule with one methyl group added, also directly acts on NMDA receptors [45]. However, it remains unknown whether DMG, with two methyl groups added to glycine, has the capacity to modulate NMDA receptors.

As enhancement of NMDA receptor function (directly or indirectly) has been associated with the therapeutic potential of sarcosine for psychiatric disorders, the present study aimed to compare the effects of srcosine and DMG on NMDA receptor function. A multi-electrode array system was used to measure the NMDA receptor-mediated excitatory field potentials (EFPs) in slice preparations of mouse medial prefrontal cortex because medial prefrontal cortex has been implicated in the processing of a wide range of cognitive and emotional stimuli and is thought to function as a central hub in the brain circuitry mediating symptoms of psychiatric disorders [31].

In our preparations, with TTX, bicuculline, and in the absence of magnesium ion, the electrophysiological signals are generated by the summed electric current flowing from NMDA receptors in the neurons in the vicinity of the electrode. The frequency of EFPs reflects the open probability of NMDA receptors. The amplitude of field potentials arises from the synchronization of neural activity. In this case the amplitude is representative of the amounts of NMDA receptors are activated simultaneously. When the open probability of the channels in the neurons is dramatically increased, the number of channels opened at the same time will be increased accordingly.

The roles of sarcosine and DMG on NMDA receptor-mediated EFPs were determined by their co-application with glutamate in the absence or presence of endogenous co-agonists glycine. The manifestation of D-cycloserine, a well-known partial agonist at the glycine binding site on NMDA receptors, in the same experimental preparation was illustrated for comparison. In addition, their effects on NMDA receptor-mediated EFPs evoked by glutamate plus D-serine, another endogenous co-agonist for the glycine site of the synaptic NMDA receptors, were assessed. Finally, the molecular docking simulations were used to evaluate the possible binding between NMDA receptor NR1 subunit and these ligands of interests, glycine, sarcosine, and DMG.

## Methods

### Animals and chemicals

Male ICR mice (8–10 weeks) were supplied from BioLASCO (Taiwan) and housed 4 to 5 per cage in a 12 h light/dark cycle (lights on 0700 h) with ad libitum access to water and food during the time the animals were in their home cages. All experiments were performed in accordance with the Republic of China animal protection law (Chapter III: Scientific Application of Animals) and approved by Institutional Animal Care and Use Committee of the National Health Research Institutes (NHRI-IACUC-10430-A).

Glycine and potassium chloride were purchased from J.T. Baker (Mallinckrodt Baker, Inc, Kentucky, USA). Sarcosine, DMG, ketamine, and 7-chlorokynurenic acid (7-CK) and other chemicals were obtained from Sigma (St Louis, MO, USA). The individual reagents were dissolved in an artificial cerebrospinalfluid (ACSF) containing (in mM) NaCl (120), KCl (3.5), CaCl_2_ (2.5), MgCl_2_ (1.2), NaHCO_3_ [24], NaH_2_PO_4_ (1.2), and D-glucose (11.5) at pH 7.4.

### Preparation of prefrontal cortex (PFC) slices for electrophysiological recordings

The brains of ICR mice were removed and immersed in an ice-cold ACSF, bubbled with a mixture of 95% O_2_/5% CO_2_. Coronal slices (300 μm) were cut from the frontal cortex (3–5.16 mm anterior to bregma) using a vibrating tissue slicer. After recovery for at least 1 h at room temperature, a single slice was transferred to the center area of the coated MED probe (Panasonic, Japan) and positioned to cover the 8 × 8 microelectrode array by a paint brush. The positioned slice was superfused at 2.0 ml/min with ACSF saturated with O_2_.

### Electrophysiological recordings

For electrophysiological recordings, the MED probe containing the brain slice was placed in a small incubator which was superfused with Mg^2+^ free-ACSF in 5% CO_2_/95% O_2_ at 25 °C and connected to the stimulation/recording component of MED8 multi-electrode array system (Panasonic, Japan). Mg^2+^-free ACSF was used to minimize Mg^2+^ block of NMDA receptors. The preparation of the multi-electrode dish has been described previously [6]. Briefly, the MED probe is an array of 64 planar microelectrodes, where each microelectrode has a size of 50 × 50 μm and is arranged in an 8 × 8 pattern. The interpolar distance in this type of probe (MED-P515A) is 150 μm. For sufficient adhesion of the brain slice to the MED probe, the surface of probe was treated with 0.1% polyethylenimine in 25 mM borate buffer for 8 h at room temperature. Then the probe surface was rinsed three times with distilled water for future experiments.

The field potentials at 8 sites in the 64 multi-electrode probe were recorded simultaneously with the multi-channel recording system at a 20 kHz sampling rate. The electrodes in the infralimbic prefrontal cortex were selected as the recording electrodes. In order to prevent the sodium channel-mediated action potential activity and the interference of inhibitory field potentials, all the experiments were performed with tetrodotoxin (TTX) (300 nM) and bicuculine (10 uM). The recording of spontaneous excitatory field potentials (EFPs) in the presence of TTX and bicuculline was first carried out to establish a stable baseline. The changes in the amplitude and frequency of NMDA receptor-mediated EFPs evoked by various ligands, which were continuously applied by adding them into the bath medium, were expressed as percentages of baseline, which were set at 100%.

### Statistical analyses

The frequency and amplitude (>0.2 mV) of field potentials were measured. All data are expressed as mean ± S.E.M. Statistical significance of the difference between groups was determined by one-way or two-way repeated measures ANOVA followed by a Student-Newman-Keuls post-hoc test. *p* < 0.05 was considered statistically significant.

### Molecular docking analysis

Glycine, sarcosine, and DMG were docked with the NMDA receptor NR1 ligand-binding core (PDB ID: 1PB7) [14] using Glide XP 6.5 [12, 13] from the program in Schrödinger (Schrödinger Suite 2014-4, Schrödinger, LLC, New York, NY, USA, 2014). The missing side chain atoms were built by using Dunbrack rotamer library [9] in UCSF Chimera (Version 1.6.2. [28], UC San Francisco, San Francisco, CA, USA, 2012). The water molecules beyond 5 Å of the crystallized glycine were removed. The 3D conformations and protonized states of ligands were generated using LigPrep 3.2 from Schrödinger (Schrödinger Suite 2014-4, Schrödinger, LLC, New York, NY, USA, 2014). The docking box was a cubic box with sides of 17.51 Å long, which centered as the crystalized glycine. To test whether the docking system is feasible for the ligand binding to NMDA receptor, the NMDA-NR1-Glycine complex (PDB ID 1PB7) was initially selected while glycine was redocked back to the complex. The docking structure of glycine was compared to its original crystallographic glycine structure. The root-mean-square distance (RMSD) between these two poses was only 1.49 Å. This result implied that the Glide docking program and docking settings used is suitable for the evaluating the possible binding between NMDA receptor NR1 and ligands of interests, glycine, sarcosine, and DMG.

## Results

### Characterization of NMDA receptor-mediated excitatory field potentials (EFPs)

**Fig. 1:**
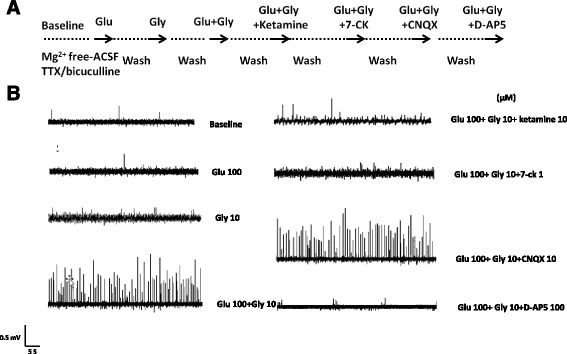
Characterization of NMDA receptor-mediated excitatory field potentials. The experimental protocol (**a**) and the representative field potential recordings (**b**) of excitatory filed potentials in an individual prefrontal cortical slice in the presence of TTX (0.3 μM) and bicuculline (10 μM) were shown after the application of glutamate (100 μM) and glycine (10 μM) alone, and then glutamate plus glycine, followed by co-application of glutamate (100 μM) plus glycine (10 μM) with ketamine (10 μM), 7-CK (1 μM), CNQX (10 μM) or D-AP5 (100 μM)

Figure 1 illustrates the experimental protocol and representative recordings of field potentials in the prefrontal cortical slices of mouse brains. The baseline of EFPs in an individual prefrontal cortical slice was initially recorded for 5–10 min in the presence of TTX and bicuculline. The glutamate (100 μM) and glycine (10 μM) alone, and then glutamate (100 μM) plus glycine (10 μM) was applied to stimulate the field potentials, followed by co-application of glutamate (100 μM) plus glycine (10 μM) with the selective NMDA receptor antagonists ketamine or D-2-amino-5-phosphonopentanoate (D-AP5), the competitive NMDA receptor glycine site antagonist 7-chlorokynurenic acid (7-CK), or 6-cyano-7-nitroquinoxaline-2,3-dione (CNQX), a competitive AMPA/kainate receptor antagonist. The baseline activity of EFPs was of low voltage under TTX treatment and the activity of inhibitory field potentials in the mouse medial prefrontal cortex was blocked in the presence of bicuculline. Perfusion of glutamate or glycine alone into the slices did not produce more EFPs than baseline, whereas co-application of glutamate (100 μM) and glycine (10 μM) significantly evoked field potentials. The frequency and amplitude of glutamate plus glycine-evoked EFPs were blocked by the NMDA receptor antagonists, ketamine and D-AP5, and the glycine binding site antagonist 7-CK, but not by AMPA/kainate receptor antagonist CNQX. These results revealed that EFPs evoked by glutamate plus glycine were mediated by NMDA receptors.

### Effects of sarcosine and DMG alone or combined with glutamate on excitatory field potentials

**Fig. 2:**
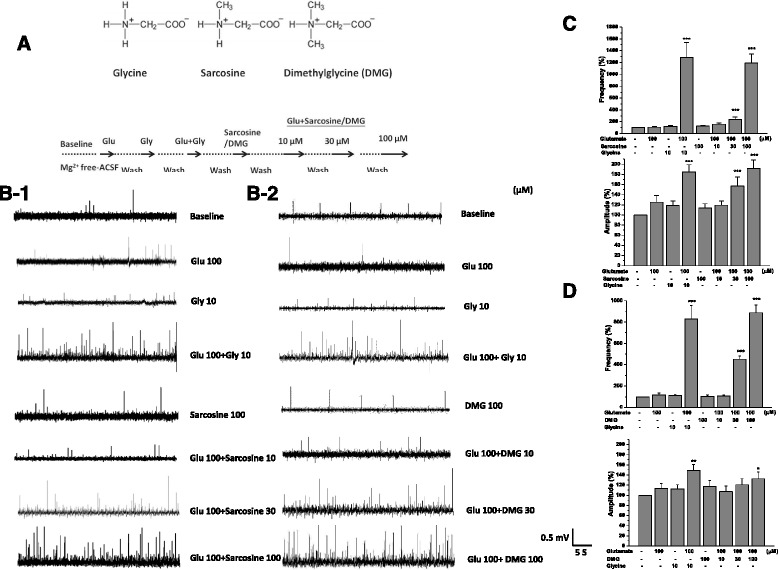
Effects of sarcosine and DMG alone or combined with glutamate on excitatory field potentials. The effects of glutamate (100 μM), glycine (10 μM), and glutamate plus glycine were examined prior to the glycine methyl derivatives to verify the slice activity. The experimental protocol (**a**), the representative field potential recordings (**b**), and the frequency and amplitude of NMDA receptor-mediated excitatory field potentials after perfusion with sarcosine or DMG (100 μM) alone and various concentrations of sarcosine ﻿(**c**)﻿﻿﻿ or DMG (﻿**d**﻿)﻿ (10, 30, and 100 μM) plus glutamate (100 μM). (Frequency: sarcosine F_7,21_ = 29.43, *p* < 0.001; DMG F_7,21_ = 48.94, *p* < 0.001; Amplitude: sarcosine F_7,21_ = 28.17, *p* < 0.001; DMG F_7,21_ = 5.28, *p* < 0.001). Data are expressed as percentage of baseline, mean ± SEM (*n* = 4). ***p < 0.001 compared with baseline

To test if DMG, like sarcosine, acts as NMDA receptor glycine binding site co-agonists, the concentration-dependent effects of these three glycine-derivatives on excitatory field potentials were examined in two conditions which are sarcosine, or DMG alone or combined with glutamate (100 μM). In each brain slice glutamate (100 μM), glycine (10 μM), and glutamate plus glycine were sequentially applied as positive controls followed by one of N-methyl glycine derivatives in the absence and presence of glutamate (Fig. 2a). Sarcosine or DMG (100 μM) alone had no effect on EFPs similar to glycine alone (Fig. 2b-c). Co-application of sarcosine or DMG (10, 30, and 100 μM) with glutamate resulted in concentration-dependent enhancement of the frequency and amplitude of the excitatory field potentials (Fig. 2).

### Effects of sarcosine and DMG on glutamate/glycine-evoked excitatory field potentials

**Fig. 3:**
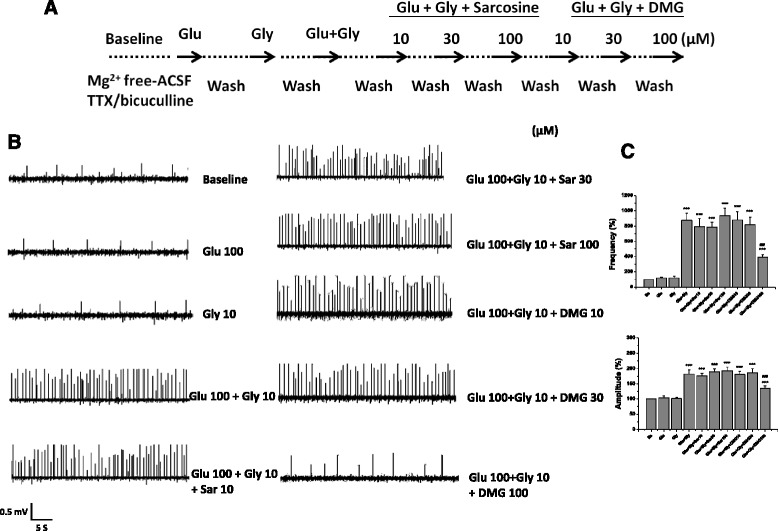
Effects of sarcosine or DMG on glutamate/glycine-evoked excitatory field potentials. The experimental protocol (**a**), the representative field potential recordings (**b**), and the frequency and amplitude of NMDA receptor-mediated excitatory field potentials (**c**) after application of glutamate, glycine, glutamate (100 μM) plus glycine (10 μM) and various concentrations (10, 30 and 100 μM) of sarcosine and DMG sequentially with glutamate plus glycine. The data were analyzed by one-way repeated measures ANOVA (Frequency: F_9,27_ = 50.58, *p* < 0.001; Amplitude: F_9,27_ = 54.64, *p* < 0.001). Data are expressed as percentage of baseline, mean ± SEM (*n* = 4). ***p < 0.001 compared with baseline. ^###^ *p* < 0.001 compared with glutamate plus glycine

Experimental protocol was illustrated in Fig. 3a. Various concentrations (10, 30 and 100 μM) of sarcosine and DMG were sequentially applied in the presence of glutamate plus glycine after glutamate (100 μM), glycine (10 μM), and glutamate plus glycine. Both the frequency and amplitude of NMDA receptor-mediated EFPs were significantly attenuated by high concentration (100 μM) of DMG, but not affected by sarcosine (Fig. 3b, c). These observations suggest that sarcosine is a co-agonist, in line with previous reports, whereas DMG acts more like a partial agonist at glycine binding site of NMDA receptors.

### Effects of D-cycloserine on NMDA receptor-mediated excitatory field potentials

**Fig. 4:**
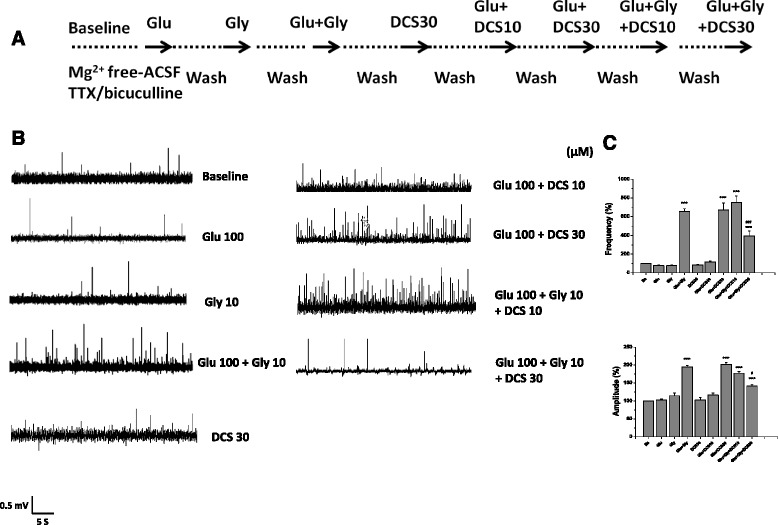
Effects of D-cycloserine on NMDA receptor-mediated excitatory field potentials. The experimental protocol (**a**), the representative field potential recordings (**b**), and the frequency and amplitude of NMDA receptor-mediated excitatory field potentials (**c**) after the application D-cycloserine alone, co-application of D-cycloserine (10 and 30 μM) with glutamate or glutamate plus glycine. The data were analyzed by one-way repeated measures ANOVA (Frequency: F_8,24_ = 70.25, *p* < 0.001; Amplitude: F_8,24_ = 100.8, *p* < 0.001). Data are expressed as percentage of baseline, mean ± SEM (*n* = 4). ***p < 0.001 compared with baseline. ^###^ *p* < 0.001 compared with glutamate plus glycine. DCS:D-cycloserine

This experiment was designed to reveal if D-cycloserine, a well-known partial agonist at the glycine binding site on NMDA receptors, produces similar effects on NMDA receptor-mediated EFPs as DMG. The protocol and representative recordings were shown in Fig. 4a and b. D-cycloserine alone did not evoke EFPs. The frequency and amplitude of EFPs were significantly increased by co-application of D-cycloserine at 30 μM, but not 10 μM, with glutamate. However, the frequency and amplitude of glutamate plus glycine-evoked EFPs were attenuated by D-cycloserine (30 μM).

### Effects of 7-CK on excitatory field potentials evoked by co-application of glutamate with sarcosine, DMG and D-cycloserine

**Fig. 5:**
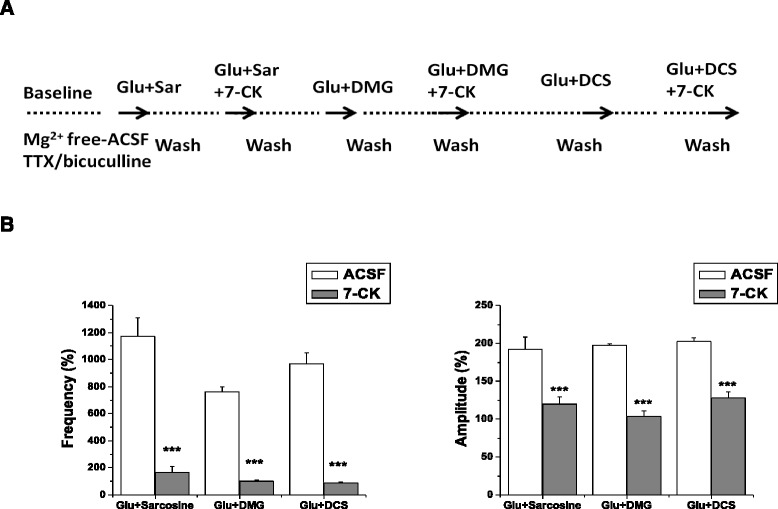
Effects of 7-CK on excitatory field potentials evoked by co-application of glutamate with sarcosine, DMG and D-cycloserine. The experimental protocol (**a**) and the frequency and amplitude of NMDA receptor-mediated excitatory field potentials (**b**) after the application of sarcosine (100 μM), DMG (100 μM), and D-cycloserine (30 μM) plus glutamate with or without 7-CK (1 μM). Two-way repeated measures ANOVA demonstrated significant effects of 7-CK (Frequency: F_1,9_ = 645.38, *p* < 0.001; Amplitude: F_1,9_ = 206.5, *p* < 0.001). Data are expressed as percentage of baseline, mean ± SEM (*n* = 4).***p < 0.001 compared with respective controls

The inhibitory effects of 7-CK, a selective antagonist at glycine binding site of NMDA receptors, on EFPs evoked by co-application of glutamate with sarcosine, DMG and D-cycloserine were assessed. As shown as previous experiments, co-application of glutamate with sarcosine (100 μM), DMG (100 μM) and D-cycloserine (30 μM) significantly increased the frequency and amplitude of EFPs. Their enhancing effects were abolished by 7-CK (Fig. 5).

### Effects of sarcosine and DMG on glutamate plus D-serine-evoked excitatory field potentials

**Fig. 6:**
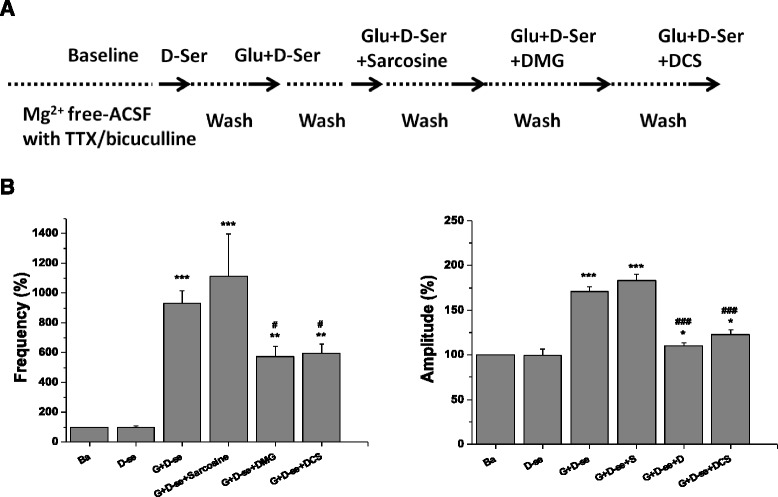
Effects of sarcosine or DMG on glutamate plus D-serine-evoked excitatory field potentials. The experimental protocol (**a**) and the frequency and amplitude of NMDA receptor-mediated excitatory field potentials (**b**) after the application of D-serine alone, D-serine plus glutamate, and co-application of sarcosine or DMG with glutamate plus D-serine were presented. The data were analyzed by one-way repeated measures ANOVA (Frequency: F_5,15_ = 39.19.43, *p* < 0.001; Amplitude: F_5,15_ = 36.79, *p* < 0.001). Data are expressed as percentage of baseline, mean ± SEM (*n* = 4). **p < 0.01, ***p < 0.01 compared with baseline. ^#^ *p* < 0.05, ^###^ *p* < 0.001 compared with glutamate plus D-serine

Glycine is a two-faceted bioactive molecule in the central nervous system [44]. In addition to being essential for the activation of NMDA receptor function, glycine is one of the main inhibitory neurotransmitters acting on glycine receptors. The simultaneous activation of excitatory NMDA receptors and inhibitory glycine receptors may interfere with the generation of excitatory field potentials. Therefore, the interactions between glycine methyl derivatives and D-serine, a selective and potent endogenous agonist for the glycine binding site of the NMDA receptors, were explored. This experiment examined the effects of sarcosine, DMG and D-cycloserine on the EFPs evoked by co-application of glutamate and D-serine. As shown in Fig. 6, D-serine alone did not affect the field potentials, whereas co-application of glutamate and D-serine evoked excitatory field potentials. The frequency and amplitude of glutamate plus D-serine-induced EFPs were significantly reduced by DMG (100 μM) and D-cycloserine (30 μM), but not sarcosine (100 μM).

### Molecular docking of glycine, sarcosine and DMG with NMDA receptors

**Fig. 7:**
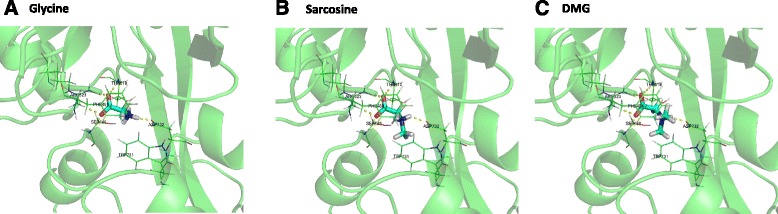
The docked poses of glycine (**a**), sarcosine (**b**), and DMG (**c**) with the NDMA receptor NR1 ligand-binding core (PDB ID: 1 PB7). Ligands were presented as cyan sticks. Potential interacting residues were presented as *green lines* and labeled with their residue codes and indices. *Yellow dashed lines* were presented for indicating potential hydrogen bonds (distance <3.5 Å)

To gain insight into the molecular interaction between glycine, sarcosine and DMG with the glycine binding site of NMDA receptor, molecular docking simulations for glycine, sarcosine, and DMG to NMDA receptor NR1 ligand-binding core (PDB ID: 1PB7) [14] were performed using Glide based on the X-ray cocrystal structure of NMDA receptor NR1 ligand-binding core with glycine as a co-crystalized ligand (PDB ID 1PB7). As shown in Fig. 7a (glycine), Fig. 7b (sarcosine), and Fig. 7c (DMG), the potential hydrogen bonds were the same: 4 hydrogen bonds which oxygen atoms at the carboxylic group were the acceptors, and the amine group as the hydrogen donor. Each oxygen atom at carboxylic group was a hydrogen bond acceptor of different guanidinium nitrogen atom of ARG523. Also, one oxygen atom was also the hydrogen bond donor of the main chain nitrogen atom of SER688. The other oxygen atom was also the hydrogen bond donor of the main chain nitrogen of THR518. Moreover, the nitrogen atom at amine group was the hydrogen donor of the side chain oxygen of ASP732. These 5 potential hydrogen bonds were the main interaction between these ligands and the binding site of NMDA receptor NR1, which were also indicated in the study of crystal structure [14]. Moreover, the nitrogen atom at amino group might form cation-pi interaction with PHE484 (glycine, sarcosine, and DMG) and TRP731 (glycine and sarcosine only).

Comparing these results, the interactions of glycine, sarcosine, and DMG to NMDA receptor were highly similar. However, the number of methyl groups bound to the amine group affected the binding affinity due to the hydrogen bonds and the nitrogen atom at the amine group which matched to our findings through EFPs.

## Discussion

The present study compared the effects of sarcosine and DMG on NMDA receptor–mediated EFPs in the mouse prefrontal cortical slices. Sarcosine and DMG alone, like endogenous co-agonists glycine and D-serine, did not change the EFPs and significantly enhanced the frequency and amplitude of EFPs in the presence of glutamate. The enhancing effects of glycine, sarcosine and DMG were blocked by the competitive NMDA receptor glycine binding site antagonist 7-CK, suggesting these two N-methyl glycine derivatives might act on the glycine binding site. From the results of molecular docking analysis, the binding modes of glycine, sarcosine, and DMG were similar, supporting that the glycine binding site is their target site. However, DMG, but not sarcosine, at higher concentration (100 μM) reduced EFPs in the presence of glutamate plus glycine/D-serine (10 μM) and the inhibitory effect of DMG could be attenuated by elevated glycine concentration (100 μM) (Additional file 1: Figure S1). Furthermore, under the same experimental procedure, the pattern of responses to D-cycloserine, a well known NMDA receptor glycine site partial agonist [22, 39], was similar to DMG. These results reveal that sarcosine is an agonist, whereas DMG acts more like a partial agonist at glycine binding site of the NMDA receptors.

Glutamate, glycine, D-serine, sarcosine, or DMG alone did not change EFPs and co-application of glutamate or NMDA (Additional file 2: Figure S2) with glycine, D-serine, or N-methyl glycine derivatives significantly increased the frequency and amplitude of EFPs. These results support the notion that occupancy of both glutamate and glycine binding sites is necessary for NMDA receptor activation. Moreover, the glutamate and glycine binding sites of NMDA receptors are scarcely occupied in the presence of TTX in acute slice preparations of medial prefrontal cortex and the NMDA receptor-mediated response observed here is independent of glutamate release.

Sarcosine enhanced EFPs with glutamate, but did not affect the glutamate plus glycine/D-serine-evoked EFPs. These results are consistent with previous findings that sarcosine enhances NMDA receptor function by directly acting as a co-agonist [45] or indirectly inhibiting the glycine transporter to increase glycine availability [17, 25]. It has been reported that sarcosine is less potent than glycine at NMDA receptor-mediated currents using whole-cell voltage clamp recordings from cultured embryonic mouse hippocampal neurons [45]. In the same manner, our data showed that in the presence of glutamate, sarcosine (100 μM) produced EFPs to the same extent as glycine (10 μM) in the prefrontal cortical slices, supporting the lower potency of sarcosine than glycine. The molecular docking analysis demonstrated that the binding modes of glycine, sarcosine and DMG are very similar. Moreover, the number of methyl groups bound to the amine group, which was the main difference between ligands, would affect the role of the nitrogen atom as the hydrogen bond donor and the binding affinity to the glycine binding site. However, the potencies of sarcosine and DMG to co-activate EFPs with glutamate in the brain slice preparations were approximately equal. It is possible that in addition to their direct interaction with NR1 subunit, there are some other factors and certain indirect effects of sarcosine and DMG contributing to their potencies to evoke EFPs with glutamate. To further elucidate the role of DMG in NMDA receptor function, it would be important to measure the whole-cell NMDA receptor-mediated synaptic currents or potential using whole-cell patch-clamp recordings.

NMDA receptors are ligand-gated ion channels assembled from NR1 and NR2 subunits. Although the glycine binding site is located in NR1 subunit, the potencies [7] and efficacies [8] of glycine binding site agonists are dependent on the NR2 subunit. In fact, NR2A- and NR2B-containing NMDA receptors are predominantly expressed in the medial prefrontal cortex [38]. Our observations in the infralimbic cortex might only reflect the effects of these N-methyl glycine derivatives on NR2A- together with NR2B-containing NMDA receptors. It remains to be investigated whether these N-methyl glycine derivatives affect NR2C- or NR2D-containing NMDA receptors in the same manner and the NR3-containing NMDA receptors that respond to glycine agonists alone [29] can be activated by N-methyl glycine derivatives directly. A study for elucidating the differential effects of these glycine analogs on NMDA receptors containing distinct types of NR2 subunits is in progress.

Modulation of NMDA receptor glycine binding site has been proposed as the next wave of drug development for schizophrenia [5], depression [10], and autism spectrum disorders [32]. It is generally believed that the augmentation of NMDA receptor transmission can improve the negative and cognitive symptoms in schizophrenia. In fact, sarcosine, when added to an existing regimen of antipsychotic drugs, has shown its efficacy for both chronically stable and acutely ill patients [18, 20, 37]. Therefore, it is postulated that NMDA receptors at key synapses are not saturated with glycine in schizophrenic patients and administration of these glycine derivatives is capable of enhancing NMDA receptor function by increasing agonist occupancy at the glycine binding site. Since DMG can be metabolized to sarcosine, it is of interest to determine if their clinical potency is higher than sarcosine in the treatment of schizophrenia.

Sarcosine can improve depression-like behaviors in rodent models and in human depression [18]. However, it may be not like NMDAR antagonist ketamine with rapid onset and sustained antidepressant effect. In fact, a glycine binding site partial agonist GLYX-13 has been reported to have long-lasting antidepressant-like effects in preclinical study and is currently in clinical trials as an add-on to already approved treatments for treatment-resistant depression patients [2, 26]. Similarly, DMG showed persistent antidepressant-like effects in mice [24].

DMG has been used as a nutritional supplement for autistic spectrum disorders and there is anecdotal evidence from parents, medical professionals, and caretakers suggesting that DMG does provide benefits for some children with autism The effectiveness of DMG combined with a large dose of vitamin B6 (pyridoxal HCl) and magnesium [43] has been revealed in young children with autism spectrum disorders although the mechanisms remain unclear. Increasing evidence indicates that dysfunction of NMDA receptors at excitatory synapses is associated with autism spectrum disorders [23]. Treatment with the NMDA receptor glycine site partial agonist D-cycloserine [42] and GLYX-13 [27, 32] rescued the deficit in the animal models. Our findings demonstrated that DMG acts as a partal agonist at glycine binding site of NMDA receptors, which might explain why it is beneficial to some children with autism.

## Conclusions

Our findings revealed that DMG might act as a partial agonist at glycine binding site of NMDA receptors and shed light on the mechanisms that might be responsible for its putative use in autism and extend their potentials in other central nervous system disorders including schizophrenia and depression.

## Additional files

Additional file 1: Figure S1. Characterization of DMG as a NMDA receptor glycine binding site partial agonist. The experimental protocol (AC) and the representative field potential recordings (BD) were shown. Increased concentration of glycine from 10 to 100 μM could surmount the inhibitory effect of DMG (100 μM). Elevated DMG concentration from 100 to 300 μM could attenuate the inhibitory effect of glycine binding site antagonist 7-CK (1 μM). (PDF 282 kb)
Additional file 2: Figures S2. Effects of sarcosine or DMG combined with NMDA and NMDA plus glycine on excitatory field potentials. The effects of glutamate (100 μM) plus glycine (10 μM) and NMDA (30 μM) plus glycine (10 μM) was compared first and blockade by MK-801. The experimental protocol (A) and the representative EFPs recordings (B) were shown as application of sarcosine (100 μM) or DMG (100 μM) combined with NMDA or NMDA plus glycine. Sarcosine and DMG produced the same effects when glutamate was replaced by NMDA. (PDF 251 kb)

## Abbreviations

7-CK: 7-chlorokynurenate
ACSF: Artificial cerebrospinal fluid
CNQX: 6-cyano-7-nitroquinoxaline-2,3-dione
DMG: N,N-dimethylglycine
EFPs: Excitatory field potentials
NMDA: N-methyl-D-aspartate
TTX: Tetrodotoxin
