# Supplementary figures and images for: Effects of sarcosine and N, N-dimethylglycine on NMDA receptor-mediated excitatory field potentials
Source: J Biomed Sci. 2017 Feb 28;24:18. doi: 10.1186/s12929-016-0314-8 (PMC5331637; doi:10.1186/s12929-016-0314-8)

# Supplement

**Fig. 1**

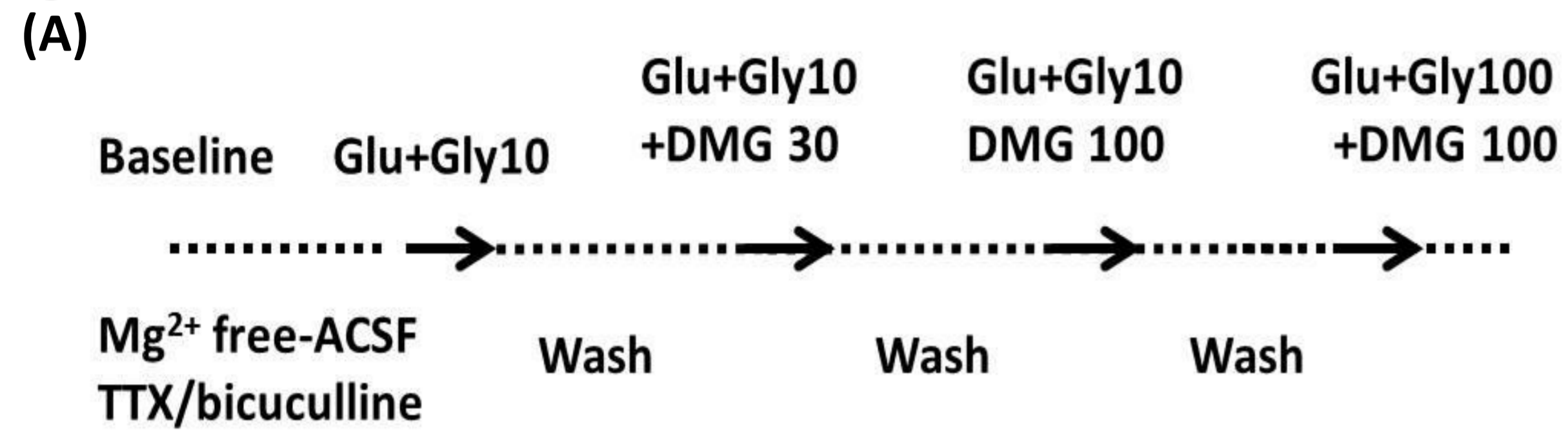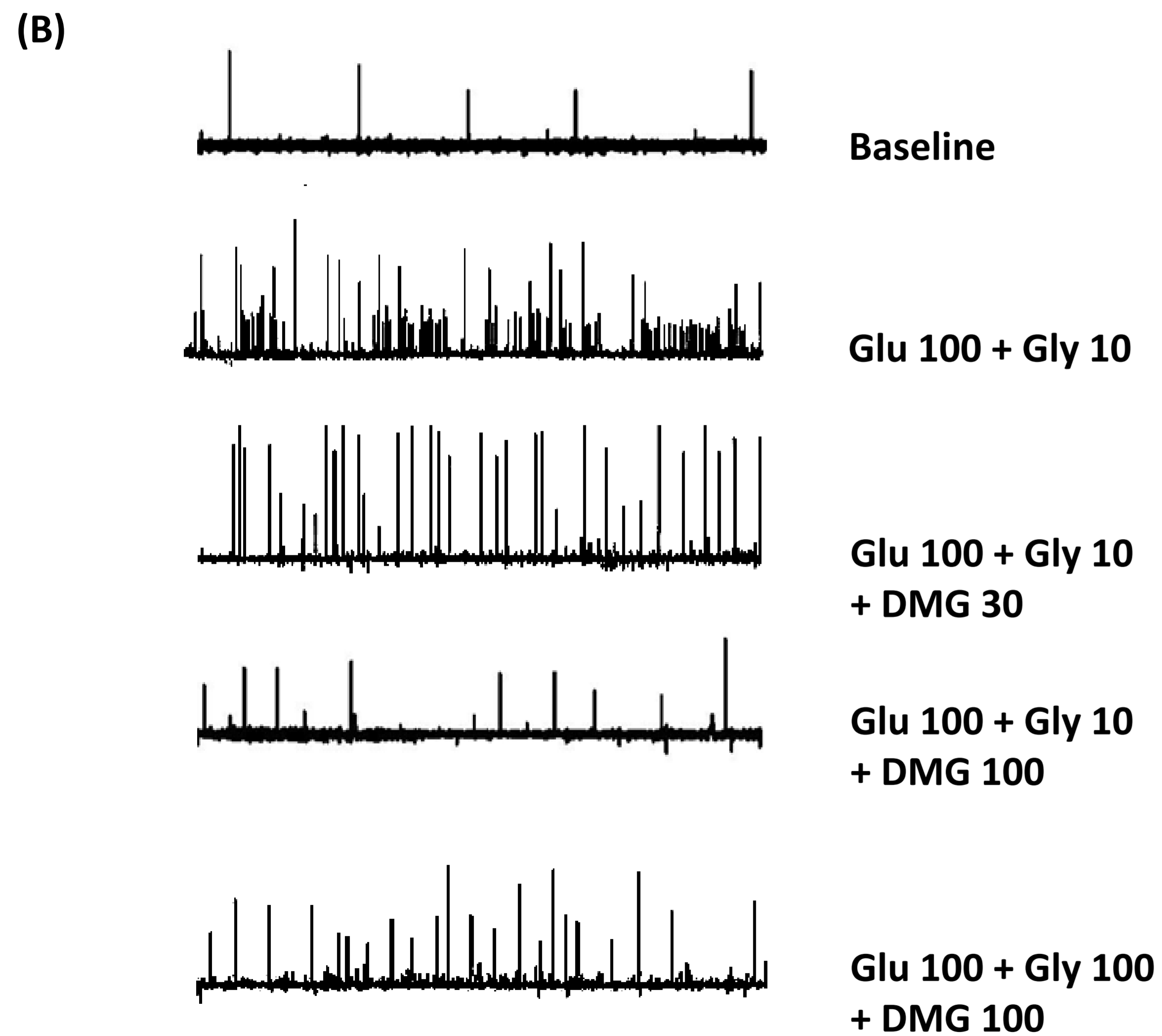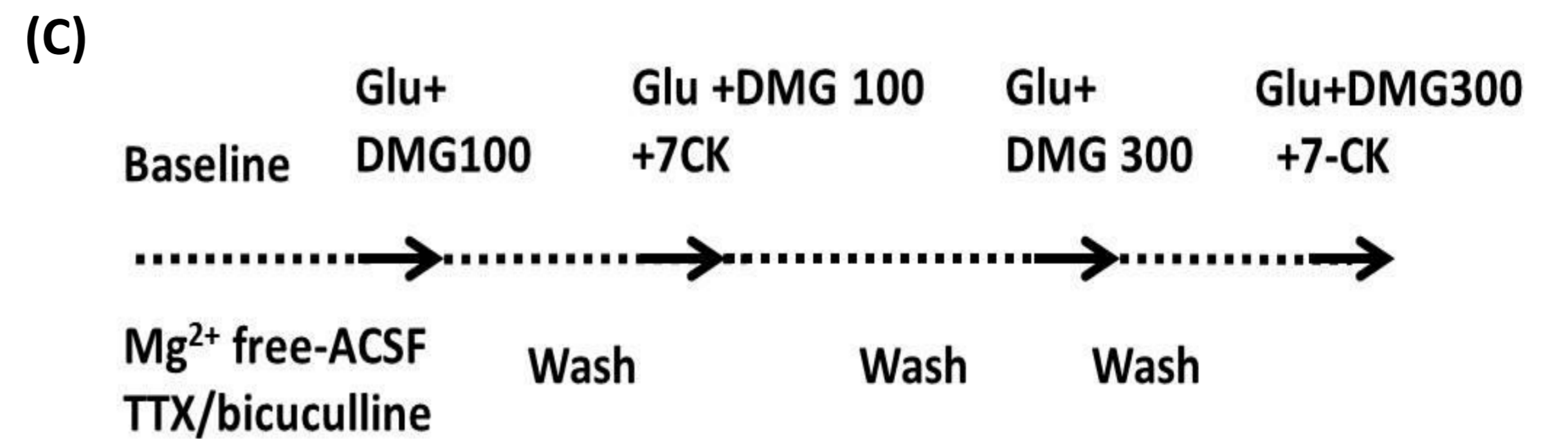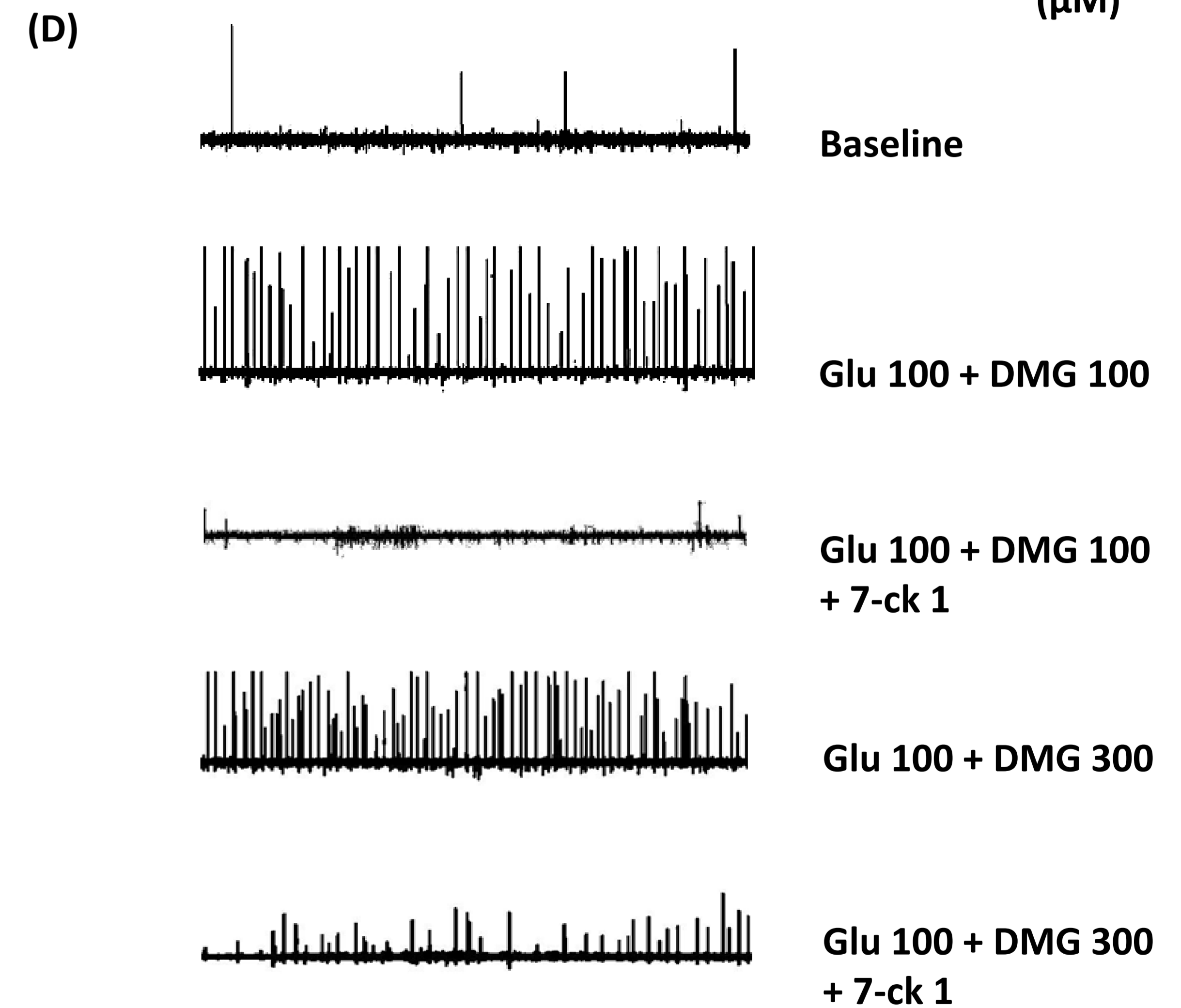

0.5 mV  
5 S

Supplement: Additional file 1: Figure S1. — Characterization of DMG as a NMDA receptor glycine binding site partial agonist. The experimental protocol (AC) and the representative field potential recordings (BD) were shown. Increased concentration of glycine from 10 to 100 μM could surmount the inhibitory effect of DMG (100 μM). Elevated DMG concentration from 100 to 300 μM could attenuate the inhibitory effect of glycine binding site antagonist 7-CK (1 μM). (PDF 282 kb) [file 12929_2016_314_MOESM1_ESM.pdf]

(A)

**Mg<sup>2+</sup> free-ACSF**  
**TTX/bicuculline**

**(B)**

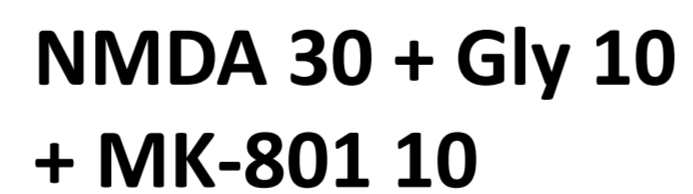

0.5 mV |  
5 s

Supplement: Additional file 2: Figures S2. — Effects of sarcosine or DMG combined with NMDA and NMDA plus glycine on excitatory field potentials. The effects of glutamate (100 μM) plus glycine (10 μM) and NMDA (30 μM) plus glycine (10 μM) was compared first and blockade by MK-801. The experimental protocol (A) and the representative EFPs recordings (B) were shown as application of sarcosine (100 μM) or DMG (100 μM) combined with NMDA or NMDA plus glycine. Sarcosine and DMG produced the same effects when glutamate was replaced by NMDA. (PDF 251 kb) [file 12929_2016_314_MOESM2_ESM.pdf]
